# A Lecture, Delivered in King's College, London, on Tuesday, the 11th of October, 1831: Being Introductory to the First Botanical Course of the Session Opening the Institution

**Published:** 1832-11

**Authors:** Gilbert T. Burnett

**Affiliations:** Professor of Botany in the College, and Fellow of several Societies.


### BOTANY.

*A Lecture, delivered in King's College, London, on Tuesday, the* 11*th of October,* 1831 : *being introductory to the first Botanical Course of the Session opening the Institution.* By Gilbert T. Burnett, Professor of Botany in the College, and Fellow of several Societies.

(Continued from page 299.)

The simple plants which constitute the so-called red snow, and rain, and hail, and dew, and which consist of one or several cellules, distinct or coadunate, give way to more advanced and regular structures in the Confervinæ, or boneworts; and these, again, to the higher grades immediately contingent, known familiarly as seaweeds, lavers, or kelp-ware. These weeds (*Thalassiophytes*, or *Phycæ*,) are followed by the land-flags, or lichens, which latter have been called *Algæ aërieæ*, to distinguish them from their aquatic allies; and, as they affect a very different station, they exhibit, as they leave the water, several important modifications of structure, to fit them for the peculiar functions they are destined to perform.

The seaweeds or wrackworts (Phycæ), including the lavers (Ulvinæ,) and the kelp-ware (Fucinæ), &c., are generally water-plants, scarcely ever growing in situations that are not frequently submerged: the lichens, on the contrary, are as universally aërial plants, affecting often peculiarly arid sites; fixing their shield-like bases on bare and barren rocks, or dead but not rapidly-decaying timber, and, when growing upon living trees, not deriving nourishment therefrom. Hence, being what are physiologically termed Epiphytes, to contradistinguish them from the true parasites, such as fungi, which not only grow upon, but draw their nourishment from, the other vegetables to which they are attached.

The marine Algæ were formerly, for the most part, included in the single genus Fucus, as the land-flags were in the single genus Lichen; but the groups of species are in both as generically distinct as in any equivalent orders of terrestrial plants; and hence they are now considered and named as such.

The aquatic flora, so long neglected that what was formerly considered knowledge can be regarded as little more than a veil for ignorance, has, by modern research, been already made a very important and interesting branch of study, and it promises to become much more so, as they well know, who ever have explored the vegetation of the sea, and all will soon confess, who, like us,

> "Shall tread from rock to rock in pleasing trance
> And note the novel forms that deck the sides
> Or float upon the surface; too fair
> Either to be divided from the place
> On which they grow, or to be left alone
> To perish in their beauty."

The history of the Fuci, as yielding iodine and kelp, two such valuable articles in medicine and commerce, affords an instructive lesson to those persons who hastily and presumptuously condemn all things as useless, the use of which they know not: for that very weed confers great benefits on man, who for ages stigmatized it as the synonyme of things most vile and worthless, useless and despised. "*Alga inutilis*," exclaimed the ancient poet; "*Vilior alga est*," in a tone of contumely, he repeats; "*Refunditur alga*," exclaims another bard: the sea itself spurns forth the worthless flag; that flag, the gathering of which for years enriched both peer and peasant on our northern coasts; the very flag that now affords the iodine which really does relieve that evil which the *manus regalis*, the boasted royal touch, (if it ever benefited the superstitious,) so long has failed to cure.

The lichens are plants familiarly known even to the least observant, as giving much of the venerable air of antiquity to aged trees, by covering their broken limbs, and reconciling the beholder to the deformity of decay. They likewise impart that subdued appearance and softness in colour and in outline which renders ancient buildings, by their calm grandeur, peculiarly impressive: hence in our language, often so admirably emphatic, they have been called "time-stains;" a name which may vie in force and elegance with any that, in any other tongue, they have hitherto obtained, and which, though long all but obsolete, may well demand its restoration to general favor.

The lichens afford several valuable dyes, a few drugs, and occasionally some food to man, though much more to certain beasts; e. g. the Cenomyce rangeriferina, or rein-deer moss, is the chief support of the Lapland herds. But the immediate uses made of these plants by us are insignificant when compared to the functions they perform in the general economy of nature. Here their utility is great, and their value may almost be stated to be in an inverse ratio to their size.

Linnæus called the Algæ, *Vernaculi,* or bond-slaves, as fettered to the rocks on which they grow. The title is particularly appropriate when applied to the lichens, which are, as it were, chained to the soil they labour to improve for the benefit of others, though from it they derive no nourishment themselves.

The first conquests of life over death, the first inroads of fertility on barrenness, are made by the smaller lichens, which, as Humboldt has well observed, labour to decompose the scorified matter of volcanoes, and the smooth and naked surfaces of sea-deserted rocks, and thus to "extend the dominion of vitality." These little plants will often obtain a footing where nothing else could be attached. So small are many, that they are invisible to the naked eye, and the decay of these, when they have flourished and passed through their transient epochs of existence, is destined to form the first exuvial layer of vegetable mould; the successive generations give successive increments to that soil from which men are to reap their harvests, and cattle to derive their food; from which forests are designed to spring, and from which future navies are to be supplied.

But how is this frail dust to maintain its station on the smooth and polished rock, when vitality has ceased to exert its influence, and the structure which fixed it has decayed? This is the point which has been too generally overlooked, and which is the most wonderful provision of all: the plant, when dying, digs for itself a grave, sculptures in the solid rock a sepulchre in which its dust may rest. For chemistry informs us that not only do these lichens consist in part of gummy matter, which causes their particles to stick together, but that they likewise form, when living, a considerable quantity of oxalic acid, which acid, when by their decay set free, acts upon the rock, and thus is a hollow formed in which the dead matter of the lichen is deposited. Furthermore, the acid, by combining with the limestone or other material of the rock, will often produce an important ingredient in the vegetable mould; and not only this, the moisture thus conveyed into the cracks and crevices of rocks and stones, when frozen, rends them, and, by continual degradation, adds more and more to the forming soil. Successive generations of these plants successively perform their duties, and at length the barren breakers, or the pumice plains of a volcano, become converted into fruitful fields.

The Fungi form a large and very curious and important class of vegetables, differing little in the lower grades from several types of Algæ; and, indeed, often considered as of inferior rank in the general scale of creation: they are simple in their structure and rapid in their growth, and, although parasitic, for the most part grow upon lifeless organic matter, which they rapidly decompose and speedily remove; thus making what has become useless to itself useful to its survivors. For these duties they are peculiarly fitted by their wandering habits; whence, by Linnæus, they were figuratively called *vagrants* or Nomades. On weak and sickly plants these parasites abound; whence they are often supposed, as blights and blasts, to produce the diseases they attend. They likewise flourish most luxuriantly amongst refuse matters, muck and offal, and often in great part form what is called mustiness, mouldiness, or mildew: hence, indeed, they have been named respectively Brands, Musts, Moulds, Mildews, Mushes, Mushrooms, &c.

The botanical term *fungi*, (a word which has now very properly become almost naturalized to our tongue,) is peculiarly expressive of the functions they perform, whether it be immediately derived from *funus* and *ago*, as indicative of their office, the removal of the dead, or intermediately from *fungor*, to discharge or execute a duty.

The natural history of these plants is one replete with interest, and, notwithstanding the little attention they commonly excite, they are constantly labouring for the general advantage. The quickness of their growth is astonishing, and the rapidity of their increase all but past belief. The Bovista, or bull-puff ball, has been computed to grow at the rate of many million cells per minute, upwards of a million per second, and to be, when at maturity, so many times larger than when beginning to germinate, that figures shrink from the expression of the sum; and Fries asserts that he has counted in a single individual plant of the smaller kinds, called smuts, ten million sporules so subtile that they rise into the atmosphere like smoke: and hence, although lost in astonishment at their prolific powers, our wonder ceases that they should be every where dispersed, and colonise every spot that affords fit nutriment for their growth.

The Fungi are associable into three chief groups, or orders; the first including those known as blights, blasts, and mildews, (Mucedines;) the second, the puff-balls, truffles, &c. (Tuberes, or Gastromyci;) and the third, the mushrooms and toadstools, (Hymenomyci, or rather Mycetes;) for it is probable that to this section the original Greek μύκης, like the modern French *champignon*, was particularly applied; for to it, in reference to the common form of the plants, both terms are peculiarly applicable; resembling, as many do, the handle of a sword, and others the pinions of a watch.

The mosses (Musci, using the term in an extended sense, though far less vaguely than formerly was done, when almost every thing moist and soft was called a moss,) will include in one class, along with the true mosses or mossworts (Muscosæ or Bryodes), the liverworts (Hepaticæ) and the stoneworts Charæ or Muscharæ), orders which, although sufficiently distinct, have several important characters in common, by which they are associated together more naturally than either with any other class. The liverworts (Hepaticæ) which now are classed with the mosses, were formerly considered more nearly connected to the flags; and, indeed, by Linnæus they were denominated Algæ hepaticæ; and the curious stoneworts (Characeæ) have likewise in general been separated too widely from their natural allies, both when arranged, as is still done by some, with the confervæ and the ferns. From the latter they are distinguished by their simply cellular structure, and from the former by the evolution of a distinct axis of growth around which central line, various processes, as leaves or branches, are arranged. These characters associate them with the Musci, which thus exhibit, by their more elaborate forms, a further stage of vegetable development.

With the mosses the first region of the vegetable reign concludes, in which the three classes, flags, or Algæ, funguses, or Fungi, and mosses, or Musci, are included; three classes which, although essentially distinct in the more highly developed and normal genera of each, are still on their confines scarcely distinguishable from each other. The simply cellular structure of the whole is their chief bond of union: in this they all agree, and future investigations will shew it to be a most important diagnostic sign: hence they have been called Cellulosæ, or Cellulares. Furthermore, the fact may be enunciated, that these vegetables are not reproduced by seeds; and hence they have been named Acotyledons, as stated in the tabular conspectus, the musts, mustallies, or mosses of our rustic dialects and older systems. In most languages it will be found that these lower tribes of plants have originally had the same or nearly similar appellations; and, that, although their names are different now, that the difference often consists in a mere modification of the original term, and that all may be traced to a common root.

This etymological evidence it would not be right at present to dilate on; but one example may not be irrelevant to shew the general impression which their most obvious characters are calculated to produce.

Our word Moss (which the Normans gave us for the elder *reet*,) is derived immediately from the Gallic *Mousse*, a term of exactly similar meaning when applied to plants, but which also signifies froth or lather, and is itself a derivation of *Mou, soft*, or loose, like the foam of the sea or the vesicles of lather; analogous to our must, or wort, given to fermenting liquors and to many similar plants. Hence also seaweeds, many of which are called sea-froth, sea-foam, sea-membranes, &c., as already shewn, are called by the French *Mousses de mer*, or sea-mosses, the Musci Marini of the older writers, the βρύον θαλάσσιον of DIOSCORIDES and PLINY. Furthermore, Fungi, or mushrooms, are named Mousserons, moss-allies, which is perhaps a contraction of Mousse ronde, or round moss; i. e. soft or puff balls.

This softness of texture and cellular formation seems to have given names to almost every section. Thus mouldiness or mustiness is called Moisissure, and Mucor,; Mildew, like our mild-dew, Serein and Sideratio; adverting to its supposed deposition from the atmosphere, or belief that the plants were star-struck: Mucedo, Muceo, and Mucus, are of nearly similar import to each other, and to our muck or slime; just as must in mustiness, and mush in mushroom, are but corruptions of moss, or *mousse;* and hence the whole series may well associate under the common names of musts, or mushes, or mush, i. e. moss-allies, technically Myc-affines. The connexions are evident in Muscus, a moss, Muces or Mousseron, and Mouceron, a mushroom, *Mousse de mer*, and βρύον θαλασσιον, sea-weeds, Mycinema, an articulate flag, &c., shew a similar affinity to be recognised by other people and in other languages besides by ourselves and in our own.

The uses of mosses are great in the general economy of nature: well have they been called by Linnæus the Servi, or ministers of nature, filling as they do and consolidating bogs, clothing mountains even to the verge of perpetual snow, and condensing the moisture of the atmosphere; thus giving origin to rills, and being the living fountains of many streams: but of their functions more hereafter.

Linnæus, who viewed nature with the kind affections of a philosopher, and the warm imagination of a poet, gave to the ferns (Filices) the figurative name of Novaccolæ, or *new settlers:* and no synonyme could more happily express their habits and general importance; for barren tracts are colonized by ferns long before many other tribes could vegetate thereon: and on sterile soils, where other plants would perish for want of food, the hardy ferns find sustenance enough; consequently in such situation they flourish and abound, unmolested by loftier and more luxuriant shrubs and trees. Ferns are truly colonists, and to fit them for the migrations they are destined to perform, it would seem as if nature, even while developing their organs of vegetation, and giving them both shrubby and arboreous stems, had considerately restrained an equivalent evolution of the reproductive system, lest they should be encumbered by weighty seeds in their successive and continued transits over large tracts of land and in crossing extensive seas.

Hence, instead of elaborate fruits and seeds, ferns, with the stems and nearly the foliage of palms, have spores little differing from those of mushrooms and of mosses. Like them, they are most prolific; for a single frond, and one fern bears many fronds, has been computed to produce upwards of a million spores.

Like the musts and their allies, so minute are these reproductive spores of ferns that their existence was for a long time doubted, and, before microscopes exposed them to our sight, this belief was common, and many references are made to it in our older writers. Shakspeare, in allusion to this, the popular opinion, observes, "we have the receipt of fern-seed, we walk invisible." The final cause of this reduced development appears to be, that such dust-like spores should be easily transportable from place to place; and hence it is that barren heaths, and coral rocks, and new-made islands, raised probably by submarine volcanoes, after that lichens and mosses have first subdued the sterility of stone, are colonized by ferns, the heralds of a more luxuriant vegetation, and harbingers of plants more immediately subservient to the purposes of man. And not only is such the course which nature now pursues in the conversion of barren into fertile soils, but geology informs us that such was the scheme of her primæval operations in preparing the earth for the reception of man; for, from the strata in which ferns are found, it is more than probable that they preceded and prepared the way for the introduction of many other vegetables, for the higher animals, and for the human race. The peculiar characteristics of the several groups of moss-like, jointless, and jointed, ferns, must, from the limits to which an introductory sketch is of necessity confined, be reserved for subsequent explanation; but this need not be regretted, for, as the illustrations successively become more and more familiar, they will of course require, in this bird's-eye view, a less and less elaborate description, and for some a mere nominal reference will probably suffice.

Hence, the grasses and the sedges, the lilies and the palms, the pines, &c., although much more important plants, as ministering to the comforts and conveniences of man, will, from their being so much more famila r to all, require *far* less descriptive detail than the lower classes of mosses, flags, and fungi; many of which are comparatively so much less known.

The grasses and sedges, though in some features similar to the shave-grass-ferns, are as characteristically distinguished by the higher development of their organs of reproduction, as the ferns are by that of their organs of nutrition. In this class it is that true flowers are first observed, and the fruit no longer developed as spores, but in the form of grains. Hence they have been called by some botanists, in reference to their fruit, Graniferæ; by others, referring to the husks within which their flowers are found, Glumaceæ; and by others again, from their stalks, which are called straws or culms, Culmiferæ.

The grain-bearing, husk-flowered, or straw-stalked plants of the botanist are the grasses and sedges of the farmer: but as these, including in the first-named the cereal species, or corn, can no longer be referred to the single genus Gramen, it has been proposed to call them collectively *Segetes*, or *Grassedges*, (or Gracarices,) to avoid the periphrases Gramina and Carices, grasses and sedges; Plantæ glumaceæ, P. graniferæ, P. culmiferæ, and so forth.

The *grasses* pass by the *reeds* and *canes* to the *palms*, the *rushes*, and the *lilies;* the Palmæ et Lilia of the Linnæan scheme, both of which are included in the Palmites of our scale.

This class, Palmites, contains some of the most curious, splendid, and majestic, plants existing, which Linnæus called the Princes *(principes)* and Patricians *(patritii)*, while he denominated the grasses the Plebeians *(plebeii)* of the vegetable kingdom. The tulip, iris, orchis, and banian types, are the pride of our gardens and conservatories; and the palms, although insignificant when grown in our largest houses, still shew, even in confinement, what majestic plants they must be when growing unrestrained in the wild luxuriance of desert nature: for some, with erect stems, attain the height of nearly two hundred feet, and others, that are climbing palms, are found of five hundred feet in length.

The three classes forming this, the second region of the vegetable reign, include plants possessing a very peculiar and characteristic structure; which, although pervading all, is more decidedly developed in the palms and arboreous ferns, than in the grasses, sedges, or any of the other sections. This structure will be hereafter fully explained, but even now the fact may be enunciated, that anatomical investigations, in the first place, shew that these plants consist not of cells alone, as in the mosses, funguses, and flags, but of tubes and cells, more or less irregularly collected into fasciculi, which are dispersed without reticulations in the leaves, and deposited centrally within the stems; that the stems are covered externally by the squamous remnants of the successive crops of leaves, just as the bulbs of a lily is by its scales; that the oldest growths are likewise, as in the bulbs of the lily, tulip, onion, hyacinth, &c., external, and the newer growths internal; so that the parts first formed are gradually forced outwards and distended, until they become so far indurated as not to yield further to pressure from within. Hence, when this hardened girth has cnce been formed, the stems never after increase in thickness, how much soever they may increase in height. From this law of evolution a very important character results; for, as the stems can never increase in girth, and the successive crops of fruit and leaves can only be supported by maintaining a communication with the roots, by successive internal deposits of adducent and reducent vessels; so when the first-formed cylinder is filled with fibres, year after year condensed, a period at length arrives when, the internal space being filled, no further deposits can take place, and the plants inevitably die.

This period is of greater or less extent in different species; but, however great it may be, a limit is fixed in early life to their duration, beyond which they cannot pass; life being to them but a preparation for death, and the very means by which they subsist render their extinction progressively more certain.

In different palms the diameter thus first formed varies, and the height to which they grow is various likewise; but to whatever extent either may proceed, the one is decidedly the limit of the other; for every additional bud or crop of leaves depositing its fibres in the centre of the stem renders the mass more dense, and more and more confirms the outer ring, which, when filled, will permit no further fibres to descend, and the plant, without redemption, dies: should, however, this cylinder be cut through, or by any means be burst, then the term of existence may become indefinitely extended: and such is the case in the Dracæna, and the celebrated one in Franqui's garden, in the island of Teneriffe, is probably six or seven hundred years of age.

The general mode of growth being thus internal, has caused the whole region of plants in which it is found to prevail to be called Endogenæ, or inside growing plants; as the previous region, from their cellular structure, were called Cellulosæ, or Telogenæ. From the term of their existence being fixed during their earliest years, which the very act of growth renders more and more inevitable, and which is strengthened by their strength, may not improbably have led the ancients to apply the name Termes to a palm-tree, as well as to the fruit branches of other plants, when plucked, and a period put to their existence: and hence the region in which this peculiar characteristic is found to prevail may be called *Term-affines,* as indicative of this, one of the most notorious diagnostic signs.

Experiments have shewn that some bulbous plants, when freely supplied with water and with abundant food, produce more leaves than flowers, and not unfrequently the blossoms entirely fail, and, until the supplies become diminished, neither flowers nor seeds are formed. This is a matter of experience, and gardeners avail themselves of this knowledge to force unwilling plants to blossom, and barren trees to bear. A somewhat similar phenomenon is observable among certain palms, and may not improbably be accounted for on similar principles. For example, the Talipot, with its majestic columnar stem, equalling in height the mainmast of a man of war, and bearing annually, through ages, its royal crown of gigantic leaves, never flowers but once; the foliage is luxuriant in the extreme, one leaf being sometimes five and thirty feet in circumference, and large enough to cover thirty or forty men; and this very luxuriance in foliage is probably the cause of its continued barrenness in flowers: but towards the close of its existence, and when the stem has become so far indurated that fresh ducts from the terminal buds cannot readily descend, when the supplies of food are curtailed, and its last efforts to live bring inevitable death, then the Corypha blossoms, and its beautiful yellow flowers, which smell so strongly that they can be perceived at a great distance, are succeeded by an abundant crop of fruit, one tree yielding enough to supply an entire country; and thus the Corypha, after having lived so long for itself, dies for its posterity.

It rarely happens among the palms, and not frequently among any of the Term-affines, that more than the central bud is developed; and hence these plants are seldom branched, and their trunks, when elevated and no longer rhizomata, are for the most part cylindrical, or nearly so. There is no absolute necessity, however, for this abortion of the lateral buds, and hence occasionally an extra one is developed, and forms a branch. In some, as the Theban doom-palm (Cucifera thebaica, or Hyphæne coriacea), two buds are naturally and equally developed, so that the stem becomes repeatedly forked.

In certain grasses likewise, as the bamboos, the stems are branched; and various exceptions to the general law are known, which hereafter will be detailed, and which circumstances render Term-affines a preferable name to Termites; the use of which, moreover, is now forbidden, from its being appropriated by zoologists to a destructive tribe of ants.

Zamiæ, or Zemiæ, names given by the ancients to the cones of firs, which, being left to open ungathered, they believed to injure the trees, and lessen the following crop, have in modern science been devoted to the designation of several curious plants, which, with their allies, the Cycases, connect, by their habits, the ferns and palms of the Term-affines with the pines and forest trees, to which, by their internal structure, they are found to be legitimately allied.

Hence this first class of the third region, including the Zamiæ and Pini, might, to avoid a periphrase, be called Za-pini, and the two orders it contains the Zamiales and Pineales.

The naked seeds and peculiarly porous wood vessels, which so closely associate these plants, otherwise, at first sight, so apparently dissimilar, are characters but comparatively a short time known; and therefore, previous to their discovery, the Cycases and Zamias were arranged by some authors with the ferns, on account of their gyrate vernation, and by others with the palms, on account of their simple stems. But the internal structure of these plants differs greatly both from the ferns and from the palms; for, although not to the fullest possible extent confirmed, still their growth and annual depositions are decidedly external; they increase in thickness, and the layers of vessels which constitute their wood are stratified each succeeding year, externally to those of the preceding; a character which is common to this region, and which forms a strong contrast with the internal growth of the Termaffines. Hence the plants in this region increase in girth as well as height, and have no determinate period assigned for their duration. These circumstances are indicated by their collective name, Crescaffines, or cress-allies, and among them will be found examples of the tallest, the largest, and the oldest vegetables existing. From the external growth and exterior deposition of wood, these plants have been likewise called Exogenæ, or outside growers, and they have also had other names indicative of other characters, to which reference shall be made hereafter.

Zamia and Cycas, neither being indigenous to this country nor naturalized to our climate, firs, larches, yews, cypresses, and cedars, are among the most familiar examples of the first class of the Crescaffines, which is distinguished from the succeeding not only by the peculiar structure of the wood, but also by the seeds being naked, i. e. not furnished with an especial covering or seed-vessel, as in the following class: hence their descriptive synonyme, Plantæ gymnospermæ; but as this term has been otherwise, though generally incorrectly used, and as it is not applicable to these plants alone, perhaps a name compounded of the appellations of the most important genera and orders it contains, may be esteemed the most fit to designate the class, such as Zapini, i. e. Zamiæ et Pini, as before observed.

Pines, which rank among our loftiest trees, are seldom known in this country to exceed a hundred feet in height, and are not to be compared with the magnificence of the New Zealand and Canadian species, which tower from one to two, and even to three hundred feet in height, maintaining at the same time a proportionable girth. One tree, indeed, I have had an account of, (but, not being a pine, it rather belongs to the succeeding class,) which grew in New South Wales, that is said to have exceeded four hundred feet in height, being higher than the cathedral of St. Paul; and we are told that an American cypress is now existing that measures above a hundred feet in girth.

